# Circulating Level of Blood Iron and Copper Associated with Inflammation and Disease Activity of Rheumatoid Arthritis

**DOI:** 10.1007/s12011-022-03148-z

**Published:** 2022-03-28

**Authors:** Huijuan Wang, Runrun Zhang, Jie Shen, Yehua Jin, Cen Chang, Mengjie Hong, Shicheng Guo, Dongyi He

**Affiliations:** 1 Department of Rheumatology, Shanghai Guanghua Hospital, Shanghai University of Traditional Chinese Medicine, Shanghai, 200052 China; 2 Department of Rheumatology, The Second Affiliated Hospital of Shandong University of Traditional Chinese Medicine, Jinan, Shandong, 250001 China; 3 Arthritis Institute of Integrated Traditional and Western Medicine, Shanghai Chinese Medicine Research Institute, Shanghai, 200052 China; 4 Department of Medical Genetics, School of Medicine and Public Health, University of Wisconsin-Madison, Madison, WI USA

**Keywords:** Rheumatoid arthritis, Copper, Iron, Zinc, Disease activity, DAS28-CRP

## Abstract

This study aims to compare the concentrations of circulating levels of iron, zinc, and copper in blood samples of rheumatoid arthritis (RA) patients which determine the correlations with inflammation and disease activity. A total of 102 RA patients and 66 healthy controls were enrolled. Circulation of iron, zinc, and copper levels in whole blood were assessed. Hemoglobin, C-reactive protein (CRP), erythrocyte sedimentation rate (ESR), anticyclic citrullinated peptide antibody (anti-CCP) levels were collected. A meta-analysis was performed to validate our findings. Single and multiple variate generalized linear regression were applied to identify the correlation between trace elements and clinical characteristics. Blood copper level was significantly higher in RA patients (*P* < 0.001), while iron and zinc levels were decreased (*P* < 0.001 and *P* = 0.02, respectively). Meta-analysis confirmed our findings for zinc (SMD =  − 1.17, *P* < 0.001) and copper (SMD = 1.24, *P* < 0.001). Copper level was positively correlated with DAS28-CRP (*r* = 0.35, *P* < 0.01), CRP (*r* = 0.45, *P* < 0.01) and ESR (*r* = 0.58, *P* < 0.01). Iron level was negatively correlated with DAS28-CRP (*r* =  − 0.37, *P* < 0.01), CRP (*r* =  − 0.46, *P* < 0.01) and ESR (*r* =  − 0.55, *P* < 0.01). Circulating blood copper was significantly higher and positively correlated with DAS28-CRP and inflammatory markers, while circulating blood iron was decreased and negatively correlated with DAS28-CRP and inflammatory markers in RA patients.

**Supplementary Information:**

The online version contains supplementary material available at 10.1007/s12011-022-03148-z.

## Introduction

Rheumatoid arthritis (RA) is a chronic, autoimmune-based disease characterized by articular inflammation and progressive joint destruction [1]. Iron, zinc, and copper are important trace elements that are essential for the regulation of metabolism and immunological functions of the human body [2]. Zinc plays an important role in the immune system by affecting both innate and adaptive immune cells [3]. Many studies have found that endogenous zinc levels can affect both the number and the function of immune cells, including macrophages, T cells, and B cells. Activation of the immune cells mentioned above can release pro-inflammatory cytokines, such as interleukin 1 (IL-1), interleukin 6 (IL-6), and tumor necrosis factor (TNF), leading to joint damage due to RA inflammation. Patients with autoimmune diseases, such as type 1 diabetes and RA, may benefit from zinc supplementation [4, 5]. Iron is an important trace element in the synthesis of hemoglobin, and iron deficiency can lead to anemia. Anemia is a common complication in RA patients and is an important extra-articular manifestation that correlates with physical disability accordingly increased mortality. The pathogenesis of anemia in RA is complex and multifactorial, perhaps due to inflammation, iron deficiency, or both [6]. Copper is also an important trace element. Most of the copper found in the human body is in a protein-bound form, such as ceruloplasmin and superoxide dismutase, which are important for antioxidant activity and maintenance of the immune system [7]. It has been reported that inflammation can increase the concentration of copper, because copper production in hepatocytes is stimulated by pro-inflammatory interleukins [8]. This study aimed to compare the concentrations of iron, zinc, and copper in RA patients with healthy individuals in order to determine their correlation with disease activity.

## Materials and Methods

### Participants

The subjects were recruited from Guanghua Hospital Precision Medicine Research Cohort (PMRC) [9] which is a hospital-based longitudinal cohort to investigate risk factors, genetic susceptibility, and pharmacogenetics for rheumatology diseases such as RA, osteoarthritis, and ankylosing spondylitis. Healthy individuals are derived from those with an annual physical exam without rheumatological disease. Currently, PMRC has enrolled > 30,000 disease patients and 10,000 healthy individuals as controls. A total of 102 patients diagnosed with RA based on the 2010 American College of Rheumatology (ACR)/European League Against Rheumatism classification criteria for RA [10] were enrolled, and 66 age- and sex-matched controls were included for comparison from PMRC [9]. Participants with other rheumatologic diseases, acute inflammatory, renal, hepatic, gastrointestinal, metabolic, or endocrine conditions were excluded.

### Data Collection

Medical history, clinical data, and physical examination findings of patients with RA, including disease course, hemoglobin, platelet count, C-reactive protein (CRP), rheumatoid factor (RF), anticyclic citrullinated peptide antibody (anti-CCP) levels, general health scores, joint swelling, and tenderness, were collected.

Fasting peripheral blood samples were collected from patients with RA and healthy controls. Iron, zinc, and copper levels in whole blood were measured by atomic absorption spectrometry using an acetylene flame. Laboratory tests, such as platelet count, hemoglobin, CRP, ESR, RF immunoglobulin G, RF immunoglobulin A, RF immunoglobulin M, and anti-CCP, were performed using standard laboratory methods.

Disease Activity Score with 28 joints using CRP (DAS28-CRP) was calculated to estimate disease activity. The 102 patients with RA were classified into two groups (57 patients with active disease vs 45 patients in remission) based on DAS28-CRP. RA patients with DAS28-CRP < 3.2 were divided into the RA remission group, while patients with DAS28-CRP ≥ 3.2 were divided into the active RA group.

### Meta-analysis

The standard mean difference (SMD) and 95% confidence interval (CI), based on the random effects model, were applied to evaluate the difference in serum levels of zinc and copper between patients with RA and healthy controls. The significance of pooled SMD between groups was assessed using the Z-test. Heterogeneity between studies was calculated according to Cochran’s Q statistic, and the I^2^ test was used to quantify the degree of inconsistency by calculating the percentage of total between-studies variation due to heterogeneity rather than chance [11]. Statistical significance was set at *P* < 0.05. Meta-analysis was conducted using the R software (R Foundation for Statistical Computing, Vienna, Austria).

### Statistical Analysis

SPSS version 21 (SPSS Inc., Chicago, IL, USA) was used for all statistical analyses, and data are expressed as the mean ± standard error of mean. A one-way analysis of variance test was used when data were normally distributed, and the variance between groups was not significantly different. Nonparametric tests were used for non-normal distributions or unequal variance data. Stepwise multiple linear regression analysis was used to analyze the relationship between disease activity and trace element concentrations and different clinical, laboratory, and other parameters. The correlation coefficient (r) was calculated to evaluate the linear association between the level of trace elements and other parameters and the disease activity of RA. Statistical significance was set at *p* < 0.05.

## Results

### Comparison of RA and Control Groups

**Fig. 1:**
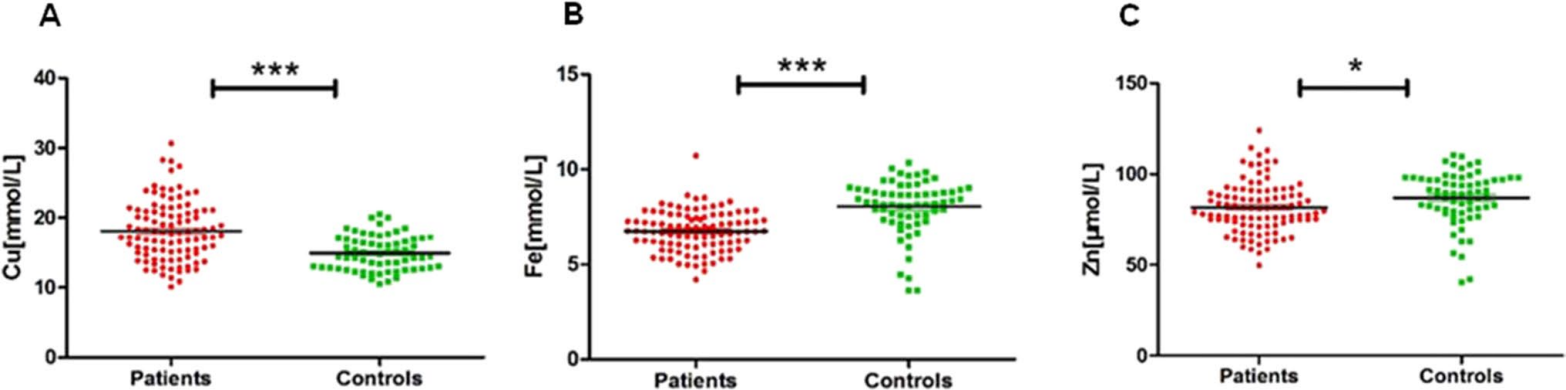
Comparison of mean blood copper, iron, and zinc between RA and control group. **A** Copper level; **B** Iron level; **C** Zinc level

In the RA group, 15 patients were men and 87 were women, while in the control group, there were 12 men and 54 women. The mean blood copper level in in the RA group was significantly higher than that of the control group (18.11 ± 4.24 µmol/L versus 14.96 ± 2.45 µmol/L, *P* < 0.001). The mean blood iron level in the RA group was 6.74 ± 1.06 mmol/L, which was significantly lower than that of the control group (8.03 ± 1.44 mmol/L, *P* < 0.001). The mean blood zinc level in the patients with RA was 81.68 ± 14.14 µmol/L, which was also significantly lower than that of the controls (87.06 ± 14.76 µmol/L, *P* = 0.02). (Table 1 and Fig. 1).

**Table 1:** Comparison between the RA and control groups

	RA group (*n* = 102)	Control group (*n* = 66)	MD	SED	P
Sex (women/men)	87/15	54/12	NA	NA	0.668
Age (years)	57.19 ± 12.06	57.95 ± 13.69	− 0.77	2.01	0.703
Copper (µmol/L)	18.11 ± 4.24	14.96 ± 2.45	3.15	0.52	< 0.001
Iron (mmol/L)	6.74 ± 1.06	8.03 ± 1.44	− 1.29	0.19	< 0.001
Zinc (µmol/L)	81.68 ± 14.14	87.06 ± 14.76	− 5.38	2.27	0.02

*MD*, mean difference; *SED*, std. error difference

### Meta-analysis of Common Trace Metals in RA

**Fig. 2:**
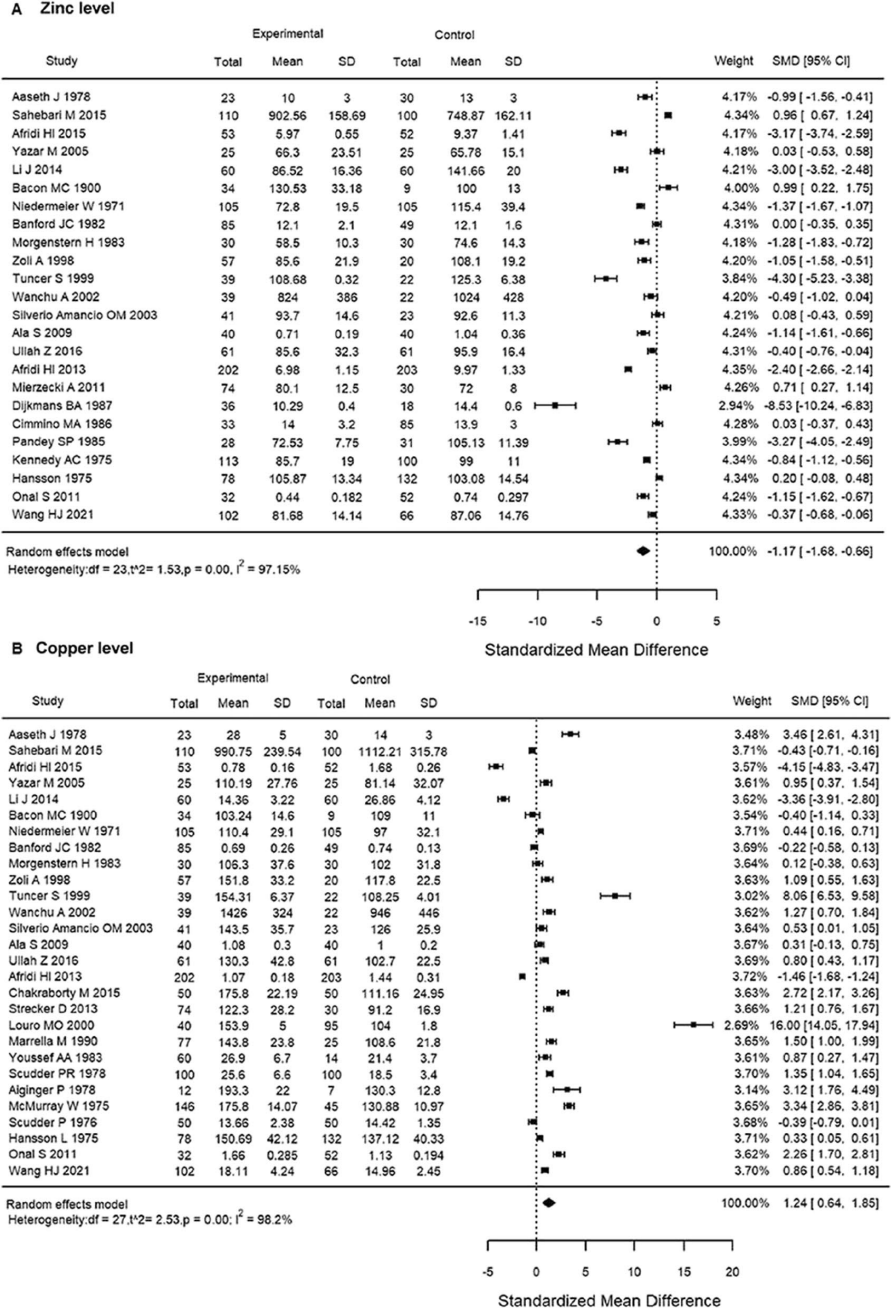
Forest plots of serum trace metal in RA patients compared with controls. **A** Zinc level; **B** Copper level

Meta-analysis of the previously published results of RA trace element levels with our results was performed. Significantly decreased zinc levels were found in patients with RA (SMD =  − 1.17, 95% *CI* =  − 1.68, − 0.66, Z =  − 4.51, *P* < 0.001). Copper was significantly higher in patients with RA than in controls (SMD = 1.24, 95% *CI* = 0.64 to − 1.85, Z = 4.04, *P* < 0.001) (Fig. 2).

### Comparison Between RA Remission, Active RA, and Control Group.

**Fig. 3:**
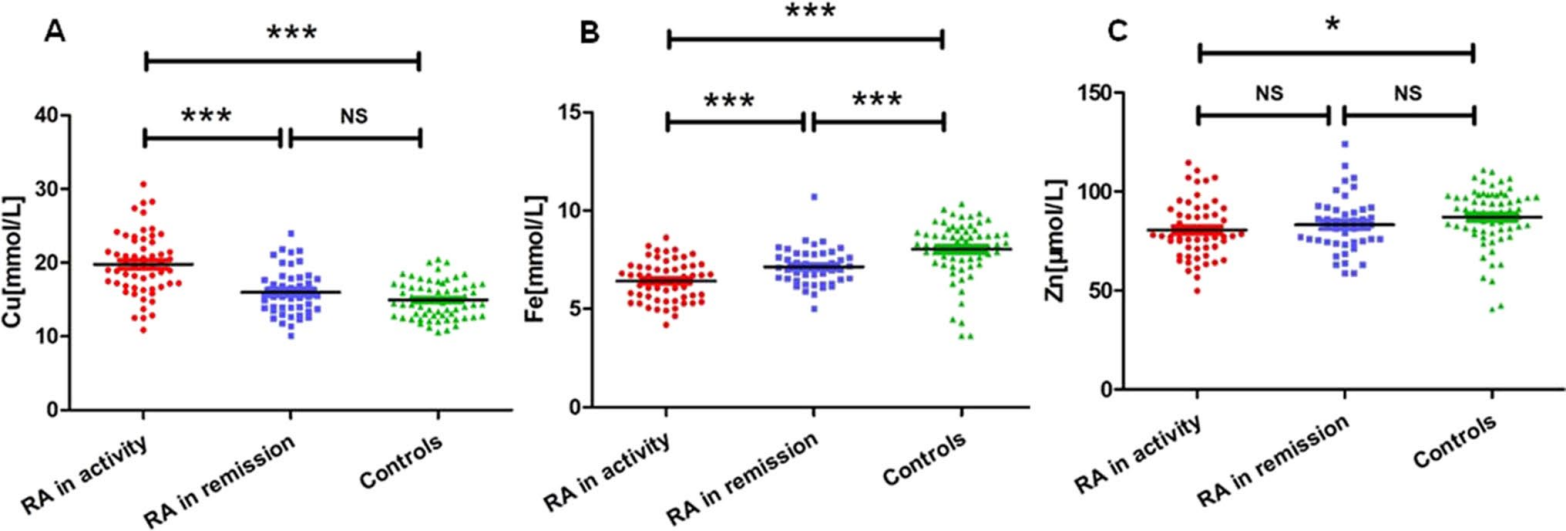
Comparison of mean blood copper, iron, and zinc between RA remission, RA activity, and control group. **A** Iron level; **B** Zinc level; **C** Copper level

Patients in the RA remission group had higher hemoglobin and iron levels, and lower platelet counts, RF-IgG, RF-IgA, and copper levels compared to those in the active RA group. There were no statistically significant differences between the two groups with respect to age, sex, disease duration, RF-IgM, anti-CCP, and zinc levels (Table 2). The mean iron level in the RA group was significantly lower than that in the control group (*P* < 0.001). However, the mean copper and zinc levels were similar, with no significant difference between the two groups (*P* = 0.058 and 0.168, respectively). A comparison of the levels of the three trace elements in the active RA group and the control group revealed that copper levels were significantly higher in patients with active RA than in controls (*P* < 0.001). The levels of iron and zinc in the active RA group were both lower than those in the control group (*P* < 0.001 and *P* = 0.014, respectively) (Table 2, Fig. 3).

**Table 2:** Copper, iron, and zinc levels of patients with RA with different disease activities

	RA		Control	*P*		
	Remission *n* = 45	Active *n* = 57	*n* = 66	Remission vs active	Remission vs control	Active vs control
Copper(mmol/L)	15.99 ± 3.21	19.79 ± 4.22	14.96 ± 2.5	< 0.001	0.058	< 0.001
Iron (µmol/L)	7.14 ± 0.96	6.42 ± 1.03	8.03 ± 1.44	< 0.001	< 0.001	< 0.001
Zinc (µmol/L)	83.18 ± 14.06	80.50 ± 14.22	87.06 ± 14.8	0.346	0.168	0.014

### Multiple Linear Regression Analysis of the Relationship Between DAS28-CRP and Trace Elements, and Other Clinical Parameters

In the multiple linear regression analysis of the relationship between disease activity (DAS28-CRP), trace elements, and other clinical parameters (sex, age, disease duration, hemoglobin, platelet count, RF-IgG, RF-IgA, RF-IgM, and anti-CCP), four items, including age, hemoglobin, copper, and RF-IgG levels, were found to be correlated with DAS28-CRP (*R*-square = 0.305) (Table 3).

**Table 3:** Multiple linear regression analysis for DAS28-CRP with the included parameters in patients with RA

Element	Variables	Coefficients	*P*
DAS28-CRP	Hemoglobin	− 0.313	0.001
DAS28-CRP	Copper	0.220	0.017
DAS28-CRP	Age	0.214	0.013
DAS28-CRP	RF-IgG	0.174	0.048

### Linear Association Between Levels of Trace Elements and Other Parameters and the Disease Activity in Patients with RA

**Fig. 4:**
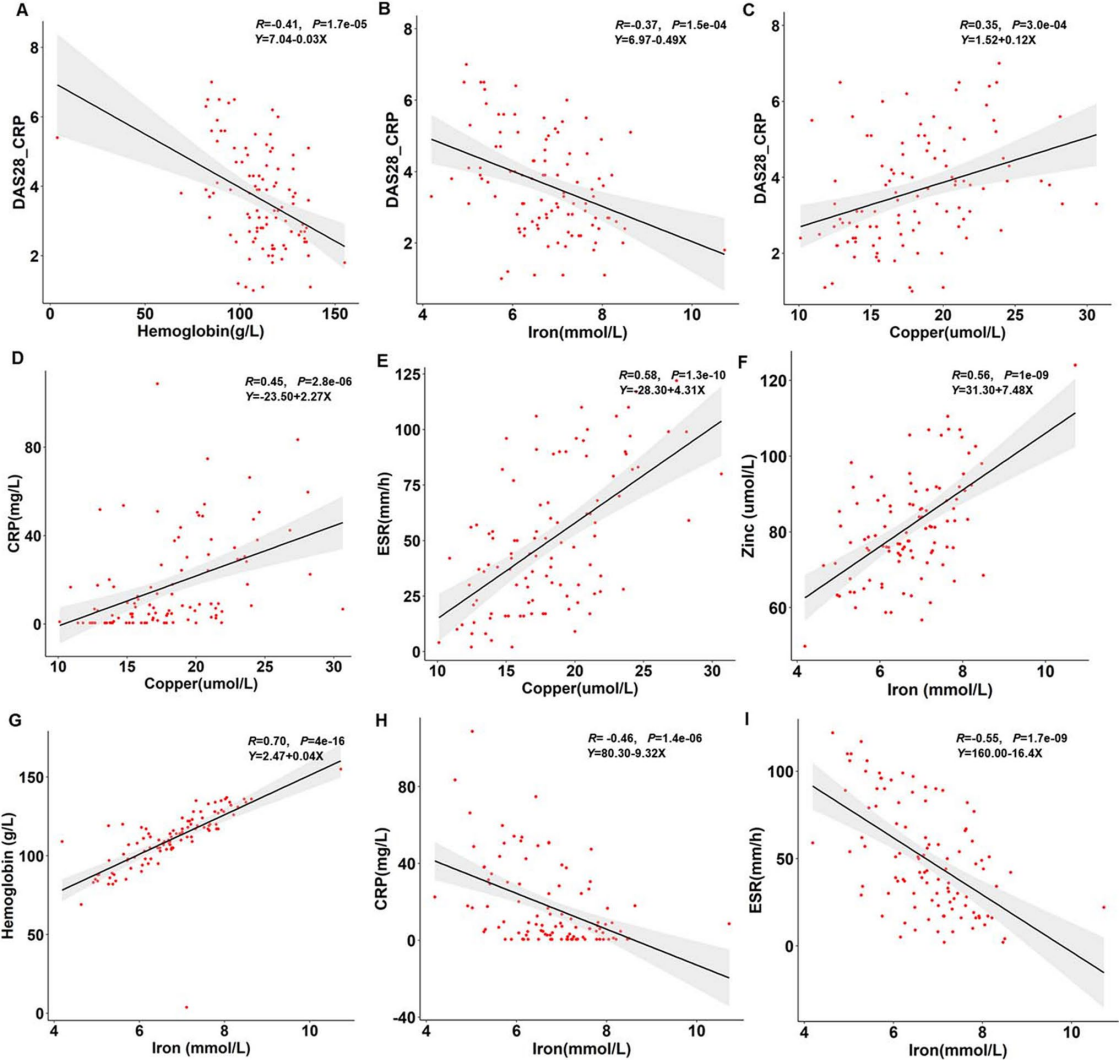
Linear association between related parameters in RA patients. **A** Linear relationship between DAS28-CRP and hemoglobin; **B** Linear relationship between DAS28-CRP and iron; **C** Linear relationship between DAS28-CRP and copper; **D** Linear relationship between copper and CRP; **E** Linear relationship between copper and ESR; **F** Linear relationship between iron and zinc; **G** Linear relationship between hemoglobin and iron; **H** Linear relationship between CRP and iron; **I** Linear relationship between ESR and iron

The linear association between the level of trace elements and other parameters and the RA disease activity showed that hemoglobin levels (*r* =  − 0.41, *P* < 0.01) and blood iron (*r* =  − 0.37, *P* < 0.01) were negatively correlated with DAS28-CRP (Supplementary Table 2, Fig. 4A, B). Blood copper level was positively correlated with DAS28-CRP (*r* = 0.35, *P* < 0.01), CRP (*r* = 0.45, *P* < 0.01) and ESR (*r* = 0.58, *P* < 0.01) (Supplementary Table 2, Fig. 4C-E). Blood iron level was highly positively correlated with blood zinc (*r* = 0.56, *P* < 0.01) and hemoglobin (*r* = 0.70, *P* < 0.01) while negatively correlated with CRP (*r* =  − 0.46, *P* < 0.01) and ESR (*r* =  − 0.55, *P* < 0.01) (Supplementary Table 2 and Fig. 4F-I).

## Discussion

This study presents a comparison of trace element levels in healthy controls and patients with RA with different disease activities. We found higher copper concentrations and lower zinc concentrations in patients with RA compared to healthy controls, confirming the reports from other investigators [12, 13]. This result was also consistent with that of Xin et al., who conducted a meta-analysis suggested that increased serum levels of copper and decreased serum levels of zinc are generally present in patients with RA [14]. These changes may be related to the antagonistic relationship between copper and zinc; overdose of zinc may reduce copper absorption, while a high dose of copper may reduce zinc absorption [15].

ESR and CRP are widely used laboratory markers of systemic inflammation [16, 17]. The result show that blood circulating copper was positively correlated with CRP (*r* = 0.45, *P* < 0.01) and ESR (*r* = 0.58, *P* < 0.01), while blood circulating iron was negatively correlated with CRP (*r* =  − 0.46, *P* < 0.01) and ESR (*r* =  − 0.56, *P* < 0.01) in RA. Copper and iron were associated with the inflammation of RA. However, ESR is under the influence of several factors, such as age, sex, anemia, and plasma proteins [18]. CRP has also been associated with the inflammation of comorbidities such as cardiovascular disease, diabetes, metabolic syndrome, pulmonary diseases, and depression [17]. DAS28-CRP and DAS28-ESR were calculated to assess the disease activity of RA [19]. The two DAS28 variants are not interchangeable especially for low activity of RA [20]. In view of CRP is more sensitive to short-time changes in inflammation [21], DAS28-CRP was used in this study.

Copper level was higher in patients with active RA compared to patients in remission and was positively associated with DAS28-CRP, a score used to evaluate the disease activity of RA. A corresponding study also reported that serum copper concentrations were positively related to disease activity, as assessed by the disease activity score [15]. Chakraborty et al. found a significant positive correlation between serum copper level, erythrocyte sedimentation rate, and morning stiffness in patients with RA [7]. Thus, serum copper level may be considered a potential biomarker of disease activity in RA. Generally, patients with RA have a high blood copper level. Penicillamine, a heavy metal (copper) chelator, is used for the treatment of RA in our hospital. It was thought that D-penicillamine may inhibit cell growth in a variety of cell types by chelating copper ions. Harada et al. suggested that D-penicillamine might help in the regression of rheumatoid synovial hyperplasia via Fas-mediated apoptosis [22].

This study showed that the blood zinc level in patients with RA, particularly those with active RA, was statistically higher than that of controls. However, there were no statistical differences between the zinc levels of patients with active RA and patients in remission, as well as between patients in remission and controls. There was no correlation between blood zinc levels and RA disease activity (*r* =  − 0.092, *P* > 0.05). This result was similar to that of a previous study conducted on juvenile idiopathic arthritis (JIA), a heterogeneous group of autoimmune diseases that arise before the age of 16 years. Hala Salah El Din Talaat et al. measured the serum levels of zinc in patients with JIA of different subtypes. They found that the mean serum zinc level in patients with JIA was significantly higher than that in the controls (*P* = 0.022). There was no statistically significant difference between the mean serum zinc levels in the JIA and control groups (*P* = 0.693) [23].

Chronic zinc deficiency in a chick model triggered a decrease in gene expression of pro-inflammatory cytokines (IL-1β, IL-6, TNF) [24]. These cytokines are vital for the pathogenesis of RA. Zinc intake increased plasma zinc concentrations and decreased CRP and IL-6 levels, when compared with a placebo [25]. Zinc plays an important role in the immune system by affects both innate and adaptive immune cells [3], although there is no strong correlation between blood zinc levels and RA disease activity.

Our study revealed that blood iron levels in patients with RA were significantly lower than those in controls, and it was negatively correlated with disease activity, which was consistent with the findings of Stefanova et al. [26]. Iron is an important trace element for the synthesis of hemoglobin, and iron deficiency can cause anemia. This study showed that blood iron levels were highly positively correlated with hemoglobin levels. Anemia in RA may be caused by the overexpression of hepcidin, which leads to local deposition of iron resulting in a decrease in iron content in peripheral blood. Studies have shown that iron can promote the production of pro-inflammatory cytokines by T cells and, thus, directly participate in the inflammatory response. Iron deposition has been observed in the central nervous system of patients with inflammatory diseases, such as multiple sclerosis, Alzheimer’s disease, and Parkinson’s disease, and in the synovial fluid of patients with RA [6, 27].

Based on the multiple linear regression analysis of the relationship between disease activity and trace element levels, and other clinical parameters, DAS28-CRP was found to be correlated with age, hemoglobin, copper, and RF-IgG levels. Except for hemoglobin, which had a negative correlation, the other three parameters all had a positive correlation with DAS28-CRP. This suggests that lower hemoglobin levels, older age, higher copper, and RF-IgG levels increase the risk of high disease activity in patients with RA. This may explain why RF-IgG can predict RA-associated consequences [28]. According to Elshafie et al. [29], RF-IgG was the strongest marker for poor prognosis because it was associated with a higher total number of affected joints. RF-IgG was associated with young age (*p* = 0.0005) and a lower age of disease onset (*P* < 0.0001), which was not found in our study.

Our study had some limitations, including the small number of subjects involved in the study and the lack of analyses of the relationship between trace elements, cytokines, and antioxidant enzymes. Further studies should focus on overcoming these limitations.

In conclusion, we found that the zinc, copper, and iron levels in the blood differed significantly in patients with RA and healthy individuals. Blood levels of iron and copper were markedly associated with RA disease activity.

## Supplementary Information

ESM 1 (DOCX 16.3 kb)
