# Supplementary material for: Circulating Level of Blood Iron and Copper Associated with Inflammation and Disease Activity of Rheumatoid Arthritis
Source: Biol Trace Elem Res. 2022 Mar 28;201(1):90–7. doi: 10.1007/s12011-022-03148-z (PMC9823016; doi:10.1007/s12011-022-03148-z)
Supplement: Supplementary file 1 — (DOCX 16.3 kb) [file 12011_2022_3148_MOESM1_ESM.docx]

**Supplementary Table2. Linear association analysis**

| **Correlations** | | | | | | | |
| --- | --- | --- | --- | --- | --- | --- | --- |
| **Correlation coefficient (r)** | Zinc | Iron | Copper | DAS28CRP | ESR | CRP | HB |
| Zinc | 1 | .559^**^ | -0.064 | -0.092 | -0.157 | -0.031 | .377^**^ |
| Iron | .559^**^ | 1 | -.364^**^ | -.367^**^ | -.552^**^ | -.457^**^ | .697^**^ |
| Copper | -0.064 | -.364^**^ | 1 | .351^**^ | .583^**^ | .445^**^ | -.331^**^ |
| DAS28CRP | -0.092 | -.367^**^ | .351^**^ | 1 | .552^**^ | .514^**^ | -.412^**^ |
| ESR | -0.157 | -.552^**^ | .583^**^ | .552^**^ | 1 | .718^**^ | -.603^**^ |
| CRP | -0.031 | -.457^**^ | .445^**^ | .514^**^ | .718^**^ | 1 | -.477^**^ |
| HB | .377^**^ | .697^**^ | -.331^**^ | -.412^**^ | -.603^**^ | -.477^**^ | 1 |
